# Author Correction: Fine cubic Cu_2_O nanocrystals as highly selective catalyst for propylene epoxidation with molecular oxygen

**DOI:** 10.1038/s41467-021-26712-y

**Published:** 2021-11-02

**Authors:** Wei Xiong, Xiang-Kui Gu, Zhenhua Zhang, Peng Chai, Yijing Zang, Zongyou Yu, Dan Li, Hui Zhang, Zhi Liu, Weixin Huang

**Affiliations:** 1 Hefei National Laboratory for Physical Sciences at the Microscale, Key Laboratory of Surface and Interface Chemistry and Energy Catalysis of Anhui Higher Education Institutes and Department of Chemical Physics, University of Science and Technology of China, 230026 Hefei, People’s Republic of China; 2 School of Power and Mechanical Engineering, Wuhan University, 430072 Wuhan, People’s Republic of China; 3 Key Laboratory of the Ministry of Education for Advanced Catalysis Materials, Zhejiang Key Laboratory for Reactive Chemistry on Solid Surfaces, Institute of Physical Chemistry, Zhejiang Normal University, 321004 Jinhua, People’s Republic of China; 4 State Key Laboratory of Functional Materials for Informatics, Shanghai Institute of Microsystem and Information Technology, Chinese Academy of Sciences, 200050 Shanghai, People’s Republic of China; 5 School of Physical Science and Technology, ShanghaiTech University, 201210 Shanghai, People’s Republic of China; 6 Dalian National Laboratory for Clean Energy, 116023 Dalian, People’s Republic of China

**Keywords:** Catalytic mechanisms, Heterogeneous catalysis, Materials for energy and catalysis

Correction to: *Nature Communications* 10.1038/s41467-021-26257-0, published online 11 October 2021.

The original version of this Article contained an error in Fig. 2, in which the units of the *y*-axis, showing the reaction rate of C_3_H_6_, were not properly displayed. In the corrected Fig. 2, the units now properly read µmol_C3H6_ h^−^^1^ g^−1^_catalyst_, and does not change any of the conclusions reported in the study. This has been corrected in both the PDF and HTML versions of the Article.

