## [Peer Review File · Nature Communications]

Title: Fine Cubic Cu₂O Nanocrystals as Highly Selective Catalyst for Propylene Epoxidation with Molecular OxygenREVIEWER COMMENTS

Reviewer #1 (Remarks to the Author):

NCOMMS-21-20906-T

The authors analyzed the propylene epoxidation reaction using molecular oxygen on nanoshaped Cu₂O catalysts and provide structure-selectivity dependent results, both experimental and theoretical. The topic is worthy of investigation, the work is performed thoroughly and reported convincingly and concisely. The level of research is high and I support publication of this work.

My specific comments:

- 1) Despite agreeing with the authors that this basic research demonstrates the effectiveness of fundamental understanding in guiding exploration of efficient catalysts for challenging heterogeneous catalytic reactions, it is the high PO productivity (high conversion as well as selectivity) that will ultimately enable epoxidation reaction with molecular O₂ at the industrial level. Consequently, propylene conversions should be also listed, to give better orientation regarding the conversions achieved and better benchmark the conversion-selectivity dependence over Cu₂O nanocubes of different sizes.
- 2) lines 72-74: The fraction of Cu(110) edge sites related to all surface Cu sites should be estimated and the appropriate numbers reported.
- 3) Lines 133-135 and Fig. S8: The CO peak area depends on surface density of sites which adsorb CO. However, also the specific surface area of the c-Cu₂O-27 and c-Cu₂O-106 samples is different, which will influence the CO peak signal. The CO signal should be normalized to surface area and then compared. Anyway, the plot will still be based on two experimental points and forced zero, which is not very convincing. More reliable results should be provided by plotting more points and observing the dependency trend.
Also, if both 110 edge and Cu₂O{100} face sites contribute to activity, and their number does not change in a linear dependency with increasing Cu₂O size, there is no guarantee the plot should be linear.

Reviewer #2 (Remarks to the Author):

In the manuscript, the authors report an investigation of reaction mechanisms and reaction sites of propylene epoxidation on Cu₂O surfaces, a system that possesses high selectivity (one of the highest ones currently), by using a combined approach between experimental and theoretical methods. A significant insight into the reaction, in particular the reaction site, has been provided based on the solid experimental work and DFT calculations. Therefore, I recommend its publication. However, the

following minor points may be considered by the authors before the publication.

1. A related work (Dai et al, PCCP 19 (2017) 25129) is not referenced, which reported that to increase the selectivity of epoxidation is to use the adsorbed O₂ molecule as an intermediate to tackle propylene directly. The work is directly relevant to the mechanisms reported in the current work and should be referenced, in my opinion.
2. In the current work, the total energies, such as chemisorption energies, reaction energies and barriers, are reported. This approach was widely used sometimes ago. Currently, many pieces of solid theoretical work reported the relevant free energies, which is better for understanding the catalytic reactions. I suggest that the relevant free energies may be mentioned in the SI.
3. In Figure 2, what do the black curves represent? They are neither explained in the figure caption nor in the main text.
4. The sentence at the beginning of page 8 is too far long and very difficult to understand completely. It should be rephrased.
5. On line 251, the authors state that "...but is around 1 eV". I do not know where 1 eV come from. My understand is that it is 1.48 eV. If this is true, it should be approximated as around 1.5 eV rather than 1 eV.

Reviewer #3 (Remarks to the Author):

In this paper the authors presented promising results for the direct epoxidation of propylene by molecular oxygen to produce PO, which is a very important reaction, and is one of the most challenging reactions in terms of selectivity. Using Cu₂O nanocrystals of different particle sizes, the authors demonstrated high PO selectivity. Results from the kinetic studies are supported by in-situ DRIFTS measurements and DFT calculations. The manuscript should be accepted for publication after the authors address the following questions:

1. The PO selectivity should be compared at comparable propylene conversions.
2. The smallest size of the three Cu₂O nanocrystals, 27 nm, shows the most promising performance. Is it possible to perform similar catalytic evaluation of Cu₂O nanocrystal with a size smaller than 27 nm.
3. For a Nature-family journal, the quality of the figures (in particular Figures 2 and 3) should be improved. For example, it is not straightforward to compare the performance of the three panels in Figure 2. The Y-axis of propylene conversion should be in the same scale (0 to 100%) to better illustrate the differences among the three catalysts. Figure 3 is too crowded. The unit of temperature is missing in the figure caption.
4. This comment might be a follow-up study if it takes too much time. It will be convincing if the authors can synthesize Cu₂O nanocrystals without any [110] facets.

5. This comment should be a follow-up study. As illustrated in Ref [12], one of the key factors for Cu is to maintain the Cu⁺ state. How does the environment at the [110] edge site affect the oxidation state of Cu? is the Cu⁺ state stable under reaction conditions? The authors should have access to synchrotron facilities at their institution. Further characterization using NEXAFS at the O K-edge, EXAFS at the Cu K-edge, or AP-XPS should be very useful in decoupling the structural and oxidation state effects for the selective epoxidation of propylene.

Author reply to Reviewer 1's comments

The authors analyzed the propylene epoxidation reaction using molecular oxygen on nanoshaped Cu₂O catalysts and provide structure-selectivity dependent results, both experimental and theoretical. The topic is worthy of investigation, the work is performed thoroughly and reported convincingly and concisely. The level of research is high and I support publication of this work.

Author reply: We appreciate the reviewer's positive recommendation and valuable comments very much. We have seriously considered the comments and revised our manuscript accordingly. We hope that the revised manuscript will be suitable for the publication.

1) Despite agreeing with the authors that this basic research demonstrates the effectiveness of fundamental understanding in guiding exploration of efficient catalysts for challenging heterogeneous catalytic reactions, it is the high PO productivity (high conversion as well as selectivity) that will ultimately enable epoxidation reaction with molecular O₂ at the industrial level. Consequently, propylene conversions should be also listed, to give better orientation regarding the conversions achieved and better benchmark the conversion-selectivity dependence over Cu₂O nanocubes of different sizes.

Author reply: We appreciate the reviewer's valuable comments very much. We definitively agree with the reviewer that the high PO productivity (high conversion as well as selectivity) will ultimately enable epoxidation reaction with molecular O₂ at the industrial level. To realize reasonable PO productivity with high PO selectivity is the ultimate target of our research on this project, and the present manuscript mainly reports that the Cu₂O{110} edge sites of finely-sized c-Cu₂O NCs can selectively catalyze propylene epoxidation with O₂ at low temperatures following a LH mechanism involving weakly-adsorbed O₂ species. Due to the use of bulk c-Cu₂O NCs with very low densities of edge sites, the C₃H₆ conversions are rather low. In the heterogeneous catalysis community, mass-specific reaction rates are considered more appropriate than conversions to report the catalytic reaction data for comparisons among different catalysts because conversions vary with the used reaction conditions such as catalyst amount, composition and flow rate of reactants but mass-specific reaction rates do not. Thus we report the mass-specific reaction rates of various c-Cu₂O NCs in catalyzing C₃H₆ oxidation with O₂ in the manuscript. But we agree with the reviewer that C₃H₆ conversions should also be reported to give the readers comprehensive information of catalytic performance of various c-Cu₂O NCs.

In reply to the reviewer, we have included the data of C₃H₆ conversions of various c-Cu₂O NCs in the revised manuscript as Supplementary Fig. 5 and described it as the following:

“As shown in Fig. 2 and Supplementary Fig. 5,”

We have also re-ordered supplementary figures in the revised manuscript accordingly.

2) lines 72-74: The fraction of Cu(110) edge sites related to all surface Cu sites should be estimated and the appropriate numbers reported.

Author reply: We appreciate the reviewer's valuable comments very much. We have calculated the density of Cu(110) edge sites and their fraction related to all surface Cu sites on various c-Cu₂O NCs based on the structural models and size distributions. The density of Cu(110) edge sites and their fraction related to all surface Cu sites were calculated as $5.517 \times 10^{18}/g_{Cu_2O}$ and 1.61% on c-Cu₂O-27, $3.93 \times 10^{17}/g_{Cu_2O}$ and 0.42% on c-Cu₂O-106, and $7 \times 10^{15}/g_{Cu_2O}$ and 0.06% on c-Cu₂O-774, respectively.

In reply to the reviewer, we have included the structural model of c-Cu₂O NCs as Supplementary Fig. 5 and the calculated density of Cu(110) edge sites and their fraction related to all surface Cu sites on various c-Cu₂O NCs as Supplementary Table 1 in the revised manuscript and described and discussed the results as the following:

“c-Cu₂O NCs are enclosed with O-terminated Cu₂O{100} faces and (Cu(I), O)-terminated Cu₂O{110} edges (Supplementary Fig. 3)^{24,29}. Based on the size distributions of various c-Cu₂O NCs, densities of Cu(110) edge sites and their fractions related to total surface Cu sites were estimated to be $6.44 \times 10^{18}/g_{Cu_2O}$ and 1.61% on c-Cu₂O-27, $4.08 \times 10^{17}/g_{Cu_2O}$ and 0.42% on c-Cu₂O-106, and $7.84 \times 10^{15}/g_{Cu_2O}$ and 0.06% on c-Cu₂O-774 (Supplementary Table 1), respectively. Surface sites of various Cu₂O NCs were probed by CO adsorption at 123 K with in situ DRIFTS (Supplementary Fig. 4). Vibrational features of adsorbed CO are barely observed for c-Cu₂O-774 NCs, but a vibrational feature at 2109 cm⁻¹ arising from CO adsorbed at the Cu(I) site²⁸ emerges for c-Cu₂O-106 NCs and grows greatly for c-Cu₂O-27 NCs. The Cu(I) sites for CO adsorption on c-Cu₂O NCs exist on the (Cu(I), O)-terminated Cu₂O{110} edges but not on the O-terminated Cu₂O{100} faces. Therefore, the density Cu₂O{110} edges are too low on large c-Cu₂O-774 NCs to be probed by CO adsorption measured with DRIFTS, but becomes high enough on fine c-Cu₂O-106 and c-Cu₂O-27 NCs.”

We have also re-ordered supplementary figures and tables in the revised manuscript accordingly.

3) Lines 133-135 and Fig. S8: The CO peak area depends on surface density of sites which adsorb CO. However, also the specific surface area of the c-Cu₂O-27 and c-Cu₂O-106 samples is different, which will influence the CO peak signal. The CO signal should be normalized to surface area and then compared. Anyway, the plot will still be based on two experimental points and forced zero, which is not very convincing. More reliable results should be provided by plotting more points and observing the dependency trend. Also, if both 110 edge and Cu₂O{100} face sites contribute to activity, and their number does not change in a linear dependency with increasing Cu₂O size, there is no guarantee the plot should be linear.

Author reply: We appreciate the reviewer's valuable comments very much. We agree that, with additional data, the plot commented by the reviewer (now Supplementary Fig. 10 in the revised manuscript) will be more convincing. Unfortunately, in our case, only c-Cu₂O-27 and c-Cu₂O-106 NCs are active at low temperatures and can be used to make the plot. Meanwhile, the (0, 0) point that we added in the plot is reasonable because no catalytic activity will appear without the active site. Moreover, the plot is used mainly to provide additional experimental results to estimate the contributions of edge and face sites of c-Cu₂O NCs to the catalytic activity at different temperatures. The C₃H₆ reaction rates of c-Cu₂O-27 and c-Cu₂O-106 NCs

vary quite linearly as a function of the peak area of CO adsorbed on their $\text{Cu}_2\text{O}\{110\}$ edge sites derived from corresponding DRIFTS results at 90 and 130 °C but not at 150 °C, suggesting that catalytic performance of c- Cu_2O -27 and c- Cu_2O -106 NCs up to 130 °C are dominantly contributed by the $\text{Cu}_2\text{O}\{110\}$ edges with the Cu(I) sites.

CO adsorbed on c- Cu_2O NCs probed by in situ DRIFTS is proportional to the amount of surface Cu(I) sites, not to the density, meanwhile, the measured C_3H_6 conversion is also proportional to the amount of active sites. Thus, a plot of C_3H_6 reaction rates of c- Cu_2O -27 and c- Cu_2O -106 NCs as a function of the peak area of CO adsorbed on their $\text{Cu}_2\text{O}\{110\}$ edge sites derived from corresponding DRIFTS results can be used to estimate the contributions of the $\text{Cu}_2\text{O}\{110\}$ edge sites on c- Cu_2O NCs to the catalytic performance.

Based on the above explanation, we believe that the data analysis and discussion in the manuscript commented by the reviewer are reasonable.

Author reply to Reviewer 2's comments

In the manuscript, the authors report an investigation of reaction mechanisms and reaction sites of propylene epoxidation on Cu_2O surfaces, a system that possesses high selectivity (one of the highest ones currently), by using a combined approach between experimental and theoretical methods. A significant insight into the reaction, in particular the reaction site, has been provided based on the solid experimental work and DFT calculations. Therefore, I recommend its publication. However, the following minor points may be considered by the authors before the publication.

Author reply: We appreciate the reviewer's positive recommendation and valuable comments very much. We have seriously considered the comments and revised our manuscript accordingly. We hope that the revised manuscript will be suitable for the publication.

1. A related work (Dai et al, PCCP 19 (2017) 25129) is not referenced, which reported that to increase the selectivity of epoxidation is to use the adsorbed O_2 molecule as an intermediate to tackle propylene directly. The work is directly relevant to the mechanisms reported in the current work and should be referenced, in my opinion.

Author reply: We appreciate the reviewer's kind suggestion very much. The paper recommended by the reviewer reports efficient C_3H_6 epoxidation with molecularly-adsorbed O_2 species on IB group metal surfaces using DFT calculations, which is highly relevant to our present work.

In reply to the reviewer, we have cited the recommended paper as Ref. 34 and discussed it in the revised manuscript as the following:

"Similar mechanisms of C_3H_6 epoxidation with molecularly-adsorbed O_2 species on IB group metal surfaces were proposed by DFT calculations³⁴."

We have also reordered all references accordingly.

2. In the current work, the total energies, such as chemisorption energies, reaction energies and barriers, are reported. This approach was widely used sometimes ago. Currently, many pieces of solid theoretical work reported the relevant free energies, which is better for understanding the catalytic reactions. I suggest that the relevant free energies may be mentioned in the SI.

Author reply: We appreciate the reviewer's valuable comments very much. We have calculated the relative free energies as suggested.

In reply to the reviewer, we have included the calculated relative free energies in the revised manuscript as Supplementary Table 1 and described the results as the following:

"DFT calculations were performed to understand the mechanisms of propylene oxidation at the $\text{Cu}_2\text{O}\{110\}$ active site (Supplementary Table 3)."

3. In Figure 2, what do the black curves represent? They are neither explained in the figure

caption nor in the main text.

Author reply: We appreciate the reviewer's careful reading very much. The black and red lines in Figure 2 represent C_3H_6 reaction rate and propylene oxide (PO), acrolein and CO_2 selectivities, respectively.

In reply to the reviewer, we have clarified this issue in the revised manuscript as the following:

“ C_3H_6 reaction rate (black) and propylene oxide (PO), acrolein and CO_2 selectivities (red)……”

4. The sentence at the beginning of page 8 is too far long and very difficult to understand completely. It should be rephrased.

Author reply: We appreciate the reviewer's kind suggestion very much.

In reply to the reviewer, the sentence commented by the reviewer has been rephrased in the revised manuscript as the following:

“Over c-Cu₂O-27 NCs (Fig. 3a), PO (m/z = 58 and 31) and CO_2 (m/z=44) productions do not appear in the C_3H_6 -TPRS profile but appear at ~80 °C with similar traces in the $C_3H_6+O_2$ -TPRS profile. Similar acrolein (m/z = 56) production traces appear at ~100 °C in both C_3H_6 -TPRS and $C_3H_6+O_2$ -TPRS profiles, and the acrolein production decreases with the temperature increasing in the C_3H_6 -TPRS profile but increases in the $C_3H_6+O_2$ -TPRS profile.”

5. On line 251, the authors state that “...but is around 1 eV”. I do not know where 1 eV come from. My understand is that it is 1.48 eV. If this is true, it should be approximated as around 1.5 eV rather than 1 eV.

Author reply: We appreciate the reviewer's careful reading very much. The “1 eV” are the calculated highest barriers of MvK mechanism for $Cu_2O\{110\}$ -catalyzed propylene epoxidation, LH and MvK mechanisms for $Cu_2O\{110\}$ -catalyzed propylene partial oxidation to acrolein, and MvK mechanism for $Cu_2O\{100\}$ -catalyzed propylene combustion. It should be “0.99-1.77 eV”.

In reply to the reviewer, we have replaced “around 1 eV” with (0.99-1.77 eV) in the revised manuscript as the following:

“, but is 0.99-1.77 eV of MvK mechanism for $Cu_2O\{110\}$ -catalyzed propylene……”

Author reply to Reviewer 3's comments

In this paper the authors presented promising results for the direct epoxidation of propylene by molecular oxygen to produce PO, which is a very important reaction, and is one of the most challenging reactions in terms of selectivity. Using Cu₂O nanocrystals of different particle sizes, the authors demonstrated high PO selectivity. Results from the kinetic studies are supported by in-situ DRIFTS measurements and DFT calculations. The manuscript should be accepted for publication after the authors address the following questions:

Author reply: We appreciate the reviewer's positive recommendation and valuable comments very much. We have seriously considered the comments and revised our manuscript accordingly. We hope that the revised manuscript will be suitable for the publication.

1. The PO selectivity should be compared at comparable propylene conversions.

Author reply: We appreciate the reviewer's valuable comment very much and agree with the reviewer. As shown in the plot of C₃H₆ conversions of various c-Cu₂O NCs (Supplementary Fig. 5 in the revised manuscript), at comparable C₃H₆ conversions, for examples, around 0.04%, c-Cu₂O-27 NCs exhibit the highest PO selectivity while c-Cu₂O-774 NCs exhibit the lowest.

In reply to the reviewer, we have discussed this issue in the revised manuscript as the following:

"Meanwhile, at comparable C₃H₆ conversions, c-Cu₂O-27 and c-Cu₂O-106 NCs exhibit much higher PO selectivities than c-Cu₂O-774 NCs. Strikingly,"

2. The smallest size of the three Cu₂O nanocrystals, 27 nm, shows the most promising performance. Is it possible to perform similar catalytic evaluation of Cu₂O nanocrystal with a size smaller than 27 nm.

Author reply: We appreciate the reviewer's kind suggestion very much. The suggestion is what we are working hard on. But we have not succeeded in synthesizing uniform c-Cu₂O NCs finer than 27 nm so far. With the findings reported in our present manuscript, we make up our mind to continue the effort on synthesis of ultrafine uniform c-Cu₂O NCs. We hope that we will succeed, but we do not know when.

3. For a Nature-family journal, the quality of the figures (in particular Figures 2 and 3) should be improved. For example, it is not straightforward to compare the performance of the three panels in Figure 2. The Y-axis of propylene conversion should be in the same scale (0 to 100%) to better illustrate the differences among the three catalysts. Figure 3 is too crowded. The unit of temperature is missing in the figure caption.

Author reply: We appreciate the reviewer's kind suggestion very much. We have re-plotted Figures 2 and 3 as suggested. Since each panels in Figure 3 are all related with reaction mechanism, we tend to combine them together. We hope that the reviewer will understand our consideration.

In reply to the reviewer, we have re-plotted Figures 2 and 3 in the revised manuscript.

4. This comment might be a follow-up study if it takes too much time. It will be convincing if the authors can synthesize Cu₂O nanocrystals without any [110] facets.

Author reply: We appreciate the reviewer's kind suggestion very much. As briefly discussed in the introduction paragraph, in our previous work (Ref. 17 in the manuscript), we studied catalytic performance of large uniform Cu₂O cubes enclosed with {100} facets, octahedra enclosed with {111} facets and rhombic dodecahedra enclosed with {110} facets and identified Cu₂O{110} facets as the active facet for propylene epoxidation with O₂, however, reaction temperatures above 150 °C were adopted due to the low density of the active site on the used large rhombic dodecahedral NCs, favoring the combustion reaction and limiting the acquired PO selectivity. The large Cu₂O octahedra not containing any {110} facets show high selectivity (around 80%) to acrolein, and the large Cu₂O cubes with very low density of {110} facets show high selectivity (around 80%) to CO₂. Inspired by these findings, we have been working on exploring highly selective Cu₂O catalysts toward PO by synthesizing uniform fine rhombic dodecahedra Cu₂O NCs with high densities of Cu₂O{110} active site, which, unfortunately, has not been realized. Later, we observed that the density of {110} edge on fine Cu₂O cubes is high enough to dominantly contribute to the catalytic activity in CO oxidation (Ref. 24 in the manuscript). Intrigued by these results, we have investigated propylene oxidation with O₂ over c-Cu₂O NCs with different sizes and report herein that fine c-Cu₂O NCs with an average size of 27 nm selectively catalyze the propylene epoxidation reaction at temperatures below 110 °C with the Cu₂O{110} edge as the active site.

We are still working hard to synthesize uniform fine rhombic dodecahedra Cu₂O NCs or very fine cubic Cu₂O NCs to explore highly selective Cu₂O catalysts for propylene epoxidation with O₂ to PO. We hope that we will succeed, but we do not know when.

5. This comment should be a follow-up study. As illustrated in Ref [12], one of the key factors for Cu is to maintain the Cu⁺ state. How does the environment at the [110] edge site affect the oxidation state of Cu? is the Cu⁺ state stable under reaction conditions? The authors should have access to synchrotron facilities at their institution. Further characterization using NEXAFS at the O K-edge, EXAFS at the Cu K-edge, or AP-XPS should be very useful in decoupling the structural and oxidation state effects for the selective epoxidation of propylene.

Author reply: We appreciate the reviewer's valuable comment very much. Following the reviewer's suggestion, we succeeded in applying for a short beamtime at the NAP-XPS end-station of Shanghai Synchrotron Radiation Facility and measured in situ NAP-XPS spectra of c-Cu₂O-27 under 0.6 mbar C₃H₆+ 0.3 mbar O₂ at temperatures up to 150 °C. The acquired Cu 2p XPS and LMM AES spectra do not show obvious oxidation of c-Cu₂O-27. However, both microscopic and spectroscopic characterization results of spent c-Cu₂O-27 NCs show that the surface of c-Cu₂O-27 NCs did not get oxidized at 90 °C but get partly oxidized at 150 °C. The discrepancy at 150 °C can be attributed to the pressure gap between NAP-XPS measurements and catalytic reaction.

In reply to the reviewer, we have added the NAP-XPS results of c-Cu₂O-27 under 0.6 mbar C₃H₆+ 0.3 mbar O₂ as Supplementary Fig. 9 in the revised manuscript and discussed this issue as the following:

“In situ NAP-XPS spectra of c-Cu₂O-27 NCs under 0.6 mbar C₃H₆+ 0.3 mbar O₂ (Supplementary Fig. 9) do not show obvious surface oxidation at temperatures up to 150 °C. The discrepancy on surface oxidation of c-Cu₂O-27 NCs at 150 °C can be attributed to the pressure gap between NAP-XPS measurements and catalytic reaction.”

“In situ C₃H₆+O₂ NAPXPS. Near-ambient pressure X-ray photoelectron spectroscopy (NAPXPS) measurements were carried out at BL02B01 of Shanghai Synchrotron Radiation Facility³⁵. The bending magnet beamline delivers soft X-ray with photon flux around 1×10^{11} photons/s, energy resolution of $E/\Delta E = 3700$ and beam spot size of $\sim 200 \mu\text{m} \times 75 \mu\text{m}$ on the sample. XPS spectra were calibrated using Au 4f_{7/2} binding energy at 84.0 eV. During the NAPXPS experiments, 0.6 mbar C₃H₆ and 0.3 mbar O₂ were introduced into the chamber, and the c-Cu₂O NCs were heated and stabilized at desirable temperatures for 0.5 h, and then the NAPXPS spectra were measured.”

We have also reordered all references and supplementary figures accordingly.

REVIEWERS' COMMENTS

Reviewer #1 (Remarks to the Author):

All my questions were answered thoroughly and the manuscript was revised accordingly. As a result, I suggest acceptance and publication of this research.

Reviewer #2 (Remarks to the Author):

I am happy with the authors's replies and changes in the manuscript. Thus, I recommend its publication.

Reviewer #3 (Remarks to the Author):

The authors have carefully and adequately addressed all my questions. I recommend the acceptance of this manuscript.

AUTHORS' REPLY TO REVIEWERS' COMMENTS

Reviewer #1 (Remarks to the Author): All my questions were answered thoroughly and the manuscript was revised accordingly. As a result, I suggest acceptance and publication of this research.

Reviewer #2 (Remarks to the Author): I am happy with the authors' replies and changes in the manuscript. Thus, I recommend its publication.

Reviewer #3 (Remarks to the Author): The authors have carefully and adequately addressed all my questions. I recommend the acceptance of this manuscript.

Author reply: We appreciate all reviewers' positive recommendation very much.