# Supplementary material for: Fine cubic Cu2O nanocrystals as highly selective catalyst for propylene epoxidation with molecular oxygen
Source: Nat Commun. 2021 Oct 11;12:5921. doi: 10.1038/s41467-021-26257-0 (PMC8505410; doi:10.1038/s41467-021-26257-0)
Supplement: Supplementary file 1 — Supplementary Information [file 41467_2021_26257_MOESM1_ESM.pdf]

Supplementary Information

Fine Cubic Cu_2O Nanocrystals as Highly Selective Catalyst for Propylene Epoxidation with Molecular Oxygen

Wei Xiong[1+], Xiang-Kui Gu[2+], Zhenhua Zhang[3], Peng Chai,[1] Yijing Zang,[4] Zongyou Yu[1], Dan Li[1], Hui Zhang,[4] Zhi Liu,[4,5] Weixin Huang[1,6] *

[1] Hefei National Laboratory for Physical Sciences at the Microscale, Key Laboratory of Surface and Interface Chemistry and Energy Catalysis of Anhui Higher Education Institutes and Department of Chemical Physics, University of Science and Technology of China, Hefei 230026, P. R. China.

[2] School of Power and Mechanical Engineering, Wuhan University, Wuhan 430072, P. R. China

[3] Key Laboratory of the Ministry of Education for Advanced Catalysis Materials, Zhejiang Key Laboratory for Reactive Chemistry on Solid Surfaces, Institute of Physical Chemistry, Zhejiang Normal University, Jinhua 321004, P. R. China.

[4] State Key Laboratory of Functional Materials for Informatics, Shanghai Institute of Microsystem and Information Technology, Chinese Academy of Sciences, Shanghai 200050, P. R. China.

[5] School of Physical Science and Technology, ShanghaiTech University, Shanghai 201210, P. R. China.

[6] Dalian National Laboratory for Clean Energy, Dalian 116023, P. R. China.

[+] These authors contributed equally.

*To whom correspondence should be addressed: huangwx@ustc.edu.cn (WH)

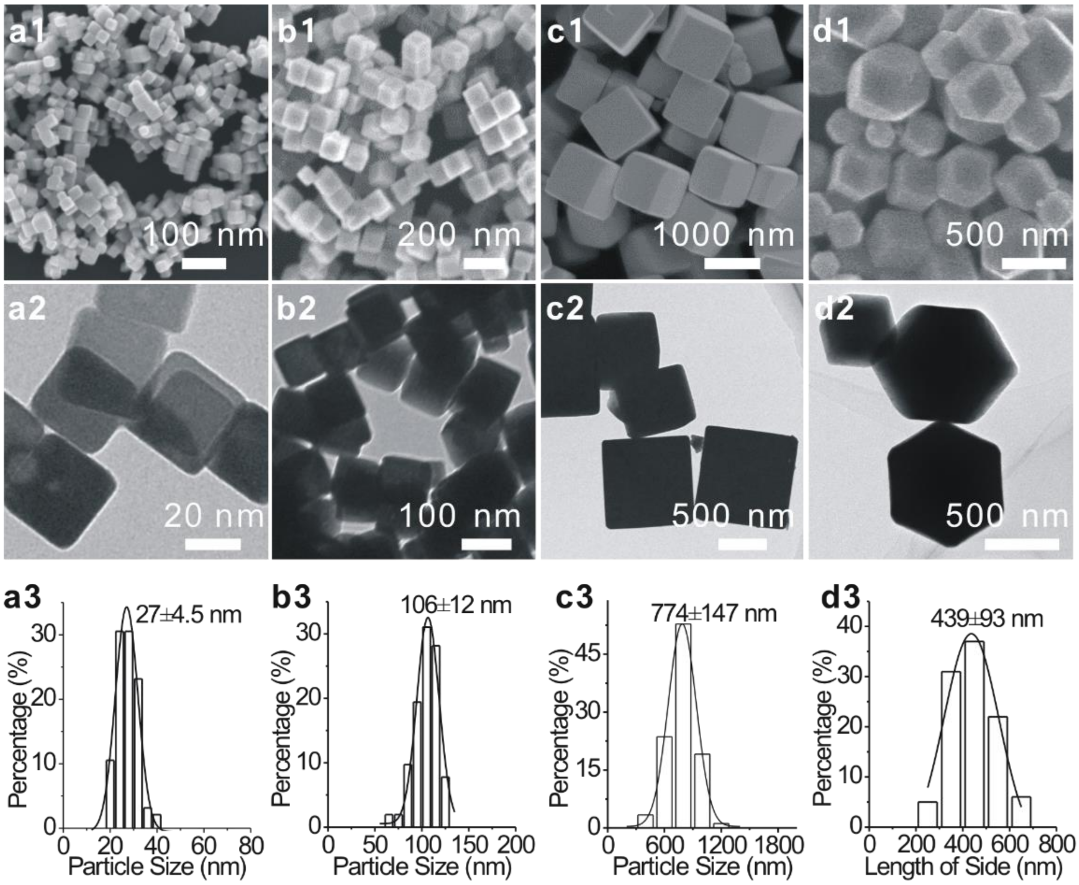

Supplementary Figure 1. SEM, TEM images and size distributions of (**a1-3**) c-Cu_2O-27, (**b1-3**) c-Cu_2O-106, (**c1-3**) c-Cu_2O-774 and (**d1-3**) d-Cu_2O-439.

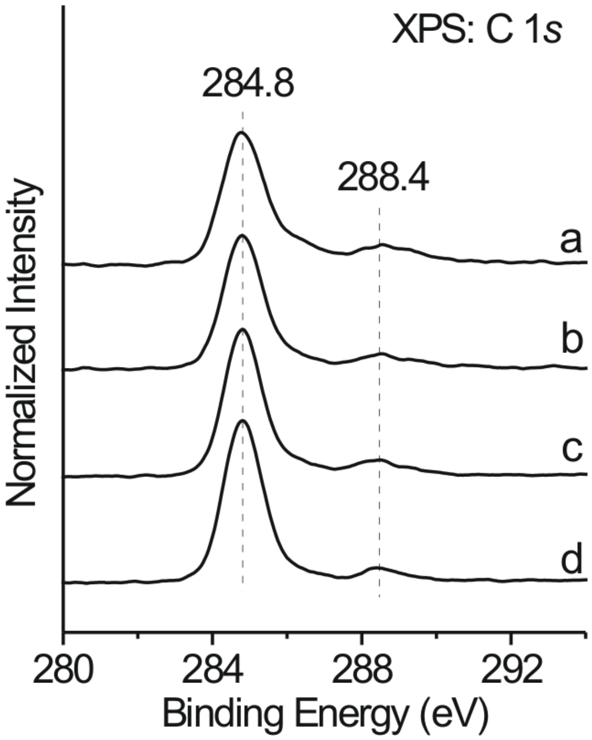

Supplementary Figure 2. A) C 1*s* XPS spectra of as-synthesized Cu_2O NCs: (a) c-Cu_2O-27, (b) c-Cu_2O-106, (c) c-Cu_2O-774, and (d) d-Cu_2O-439. C 1*s* XPS signals at 284.8, 288.4 eV can be ascribed to the adventitious carbon and various types of carbonate species.

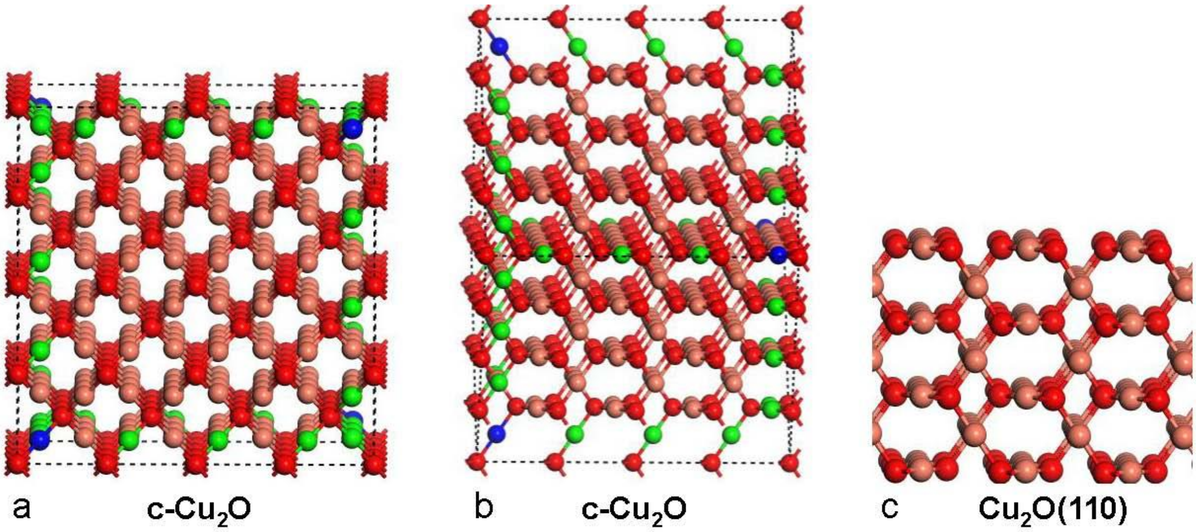

Supplementary Figure 3. Side (a) and edge (b) view of the structure of (4×4×4)-c-Cu_2O and side view of Cu_2O(110) (c). The orange, red, green, and blue spheres represent Cu atom, O atom, Cu atom on the edge, and Cu atom shared by three edges, respectively. The Cu and O atoms on the edges of c-Cu_2O NCs exhibit the same coordination structures as those on the Cu_2O{110} surfaces.

Supplementary Table 1. Calculated densities of Cu(110) edge sites and their fractions related to total surface Cu sites on various c-Cu_2O NCs.

	c-Cu_2O-27	c-Cu_2O-106	c-Cu_2O-774
Fraction of Cu{110} edge sites related to total surface Cu atoms	1.61%	0.42%	0.06%
Density of Cu(110) edge sites (g^{-1})	6.44×10^{18}	4.08×10^{17}	7.84×10^{15}

Defining L (in nm) and a (a = 0.427 nm) as the edge length and lattice parameter of a c-Cu_2O NC, the number of Cu_2O unit cell is $(L/a)^3$, and each Cu_2O unit cell contains 4 Cu atoms, thus the total Cu atom number (n) of a c-Cu_2O NC with an edge length of L can be calculated as:

$$n = 4(\frac{L}{a})^3 \quad (1)$$

The number of Cu atoms on each edge is L/a, and 4 Cu corner atoms are shared by three edges. Thus, the total number of Cu(110) edge sites (n_{edge}) of a c-Cu_2O NC with an edge length of L can be calculated as:

$$n_{\text{edge}} = 12\frac{L}{a} - 8 \quad (2)$$

The number of Cu_2O surface unit cell is $(L/a)^2$, and each Cu_2O surface unit cell contains 2 Cu atoms, thus the Cu surface atom number of each face of a c-Cu_2O NC with an edge length of L can be calculated as $2(\frac{L}{a})^2$. On each of the two basal planes of a c-Cu_2O NC, the number of Cu surface atoms is $2(\frac{L}{a})^2$; on each of the two separated lateral planes (not sharing Cu atoms with each other), there are two Cu rows shared by the basal plane, and the number of Cu surface atoms is $2(\frac{L}{a})^2 - 2\frac{L}{a}$; on each of the last two lateral planes, except for sharing two Cu rows with the basal plane, it also shares one Cu atom in each Cu row with the two separated lateral planes, and the number of Cu surface atoms is $2(\frac{L}{a})^2 - 4\frac{L}{a} + 2$. Thus, the total number of Cu surface atoms ($n_{surface}$) of a c-Cu_2O NC with an edge length of L can be calculated as:

$$n_{\text{surface}} = 12(\frac{L}{a})^2 - 12\frac{L}{a} + 4 \quad (3)$$

Thus, the fraction (f) of Cu{110} edge sites related to total surface Cu atoms of a c-Cu_2O NC with an edge length of L can be calculated as:

$$f = \frac{n_{\text{edge}}}{n_{\text{surface}}} \quad (4)$$

The density of Cu_2O is 6 g/cm^3, thus the particle number (N) of 1g c-Cu_2O NCs with an edge length of L can be calculated as:

$$N = \frac{1}{6L^3}\times10^{21} \quad (5)$$

And the density of Cu(110) edge sites (N_{edge}) of a c-Cu_2O NC with an edge length of L can be calculated as:

$$N_{\mathrm{edge}} = \frac{1}{6L^3} \times 10^{21} \times (12\frac{L}{a} - 8) \qquad (6)$$

For our c-Cu_2O NCs with size distributions shown in Figure 1, defining i as c-Cu_2O NCs with a specific edge length, x_i as the fraction of c-Cu_2O NCs with a specific edge length, fi as the fraction of Cu{110} edge sites related to total surface Cu atoms of a c-Cu_2O NC with a specific edge length, and $N_{edge,i}$ as the density of Cu(110) edge sites of a c-Cu_2O NC with a specific edge length,

Then the fraction of Cu{110} edge sites related to total surface Cu sites of our c-Cu_2O NCs can be calculated as $\sum_i x_i f_i$

The particle number of our c-Cu_2O NCs with an average edge length of L can be calculated as:

$$N = \frac{1}{6L^3} \times 10^{21} \qquad (7)$$

The density of Cu{110} edge sites of our c-Cu_2O NCs can be calculated as $N \sum_i x_i \, n_{edge,i}$

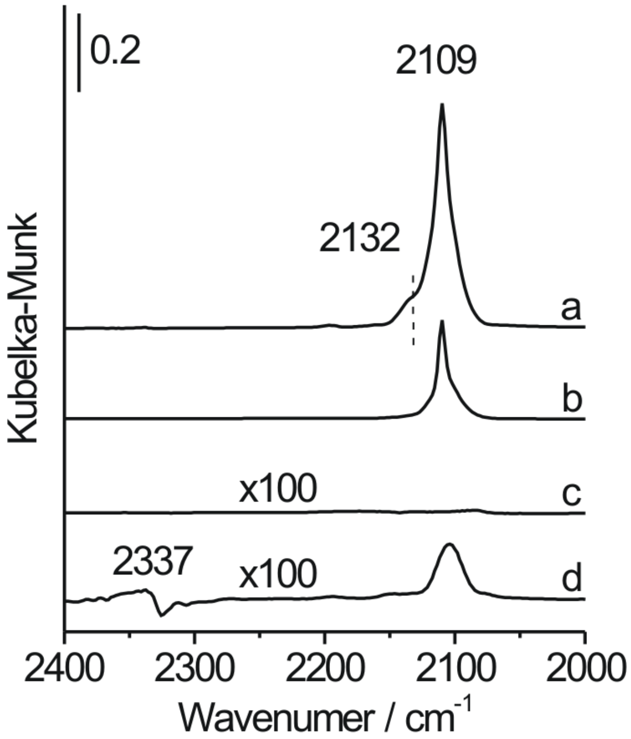

Supplementary Figure 4. In situ DRIFTS spectra of CO adsorption (P_{CO}=250 Pa) on the Cu_2O NCs at 123 K: (a) c-Cu_2O- 27, (b) c-Cu_2O-106, (c) c-Cu_2O-774, and (d) d-Cu_2O-439.

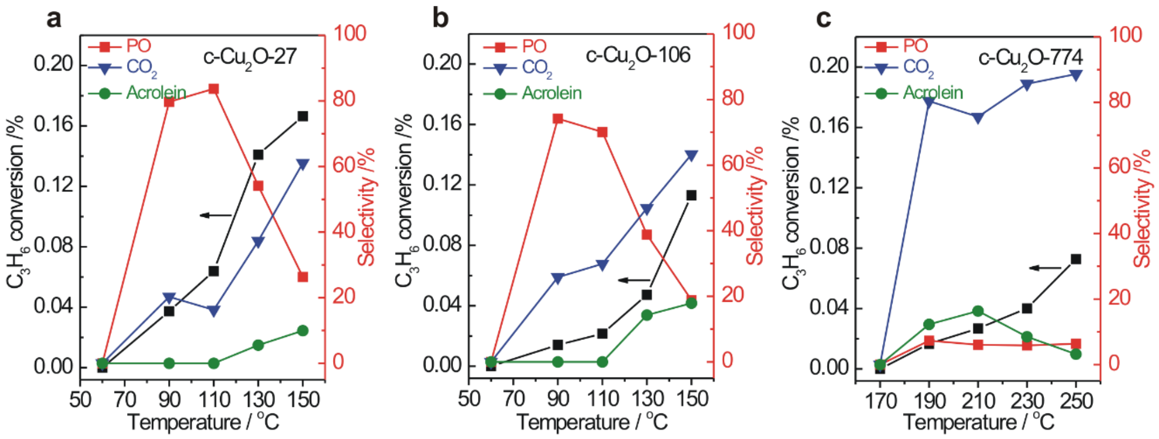

Supplementary Figure 5. C_3H_6 conversion and propylene oxide (PO), acrolein and CO_2 selectivities of the C_3H_6 oxidation with O_2 catalyzed by (**a**) c-Cu_2O-27, (**b**) c-Cu_2O-106 and (**c**) c-Cu_2O-774 NCs. Reaction condition: 200 mg catalyst, 8% C_3H_6 and 4% O_2 balanced with Ar at a flow rate of 50 mL min^{-1}.

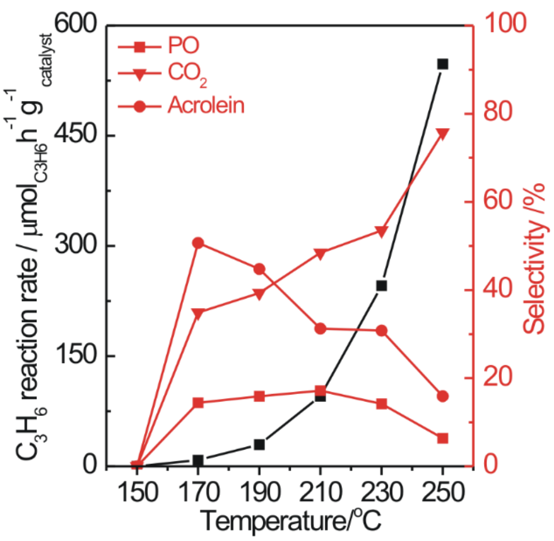

Supplementary Figure 6. C_3H_6 reaction rate and propylene oxide (PO), acrolein and CO_2 selectivities of the C_3H_6 oxidation with O_2 catalyzed by d-Cu_2O-493 NCs. Reaction condition: 200 mg catalyst, 8% C_3H_6 and 4% O_2 balanced with Ar at a flow rate of 50 mL min^{-1}.

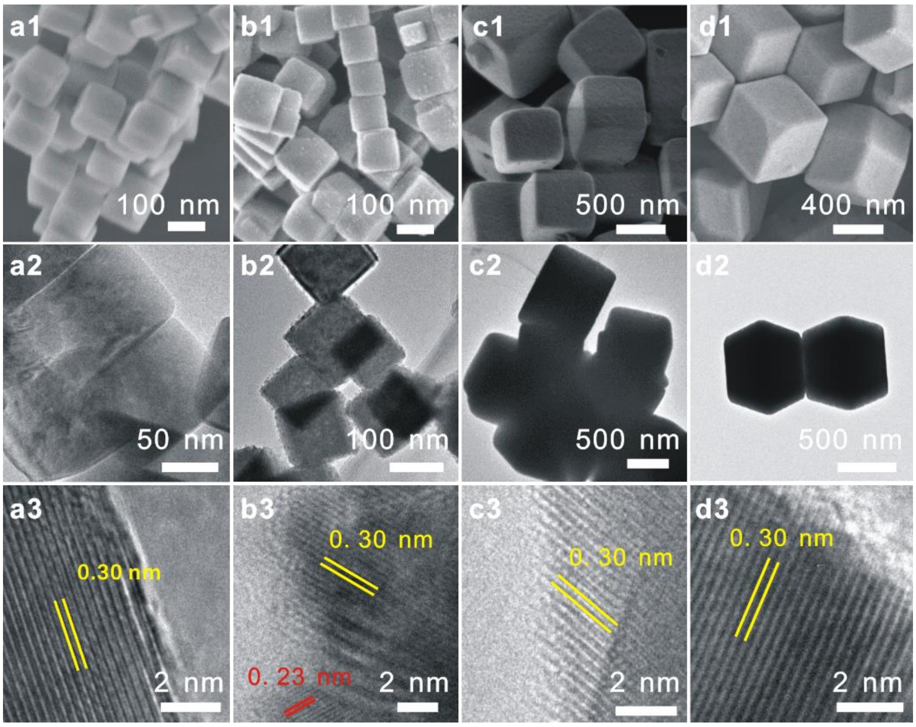

Supplementary Figure 7. SEM, TEM, and HRTEM images of typical Cu_2O NCs evaluated after propylene oxidation: (**a1**)–(**a3**) c-Cu_2O-106 at 90 °C, (**b1**)–(**b3**) c-Cu_2O-106 at 150 °C, (**c1**)-(**c3**) c-Cu_2O-774 and (**d1**)-(**d3**) d-Cu_2O-439 at 210 °C. The lattice fringes of 0.30 and 0.23 nm correspond to the spacing of the Cu_2O\{110\} (JCPDS card No. 78-2076) and CuO\{111\} (JCPDS card No. 89-5899) crystal planes, respectively.

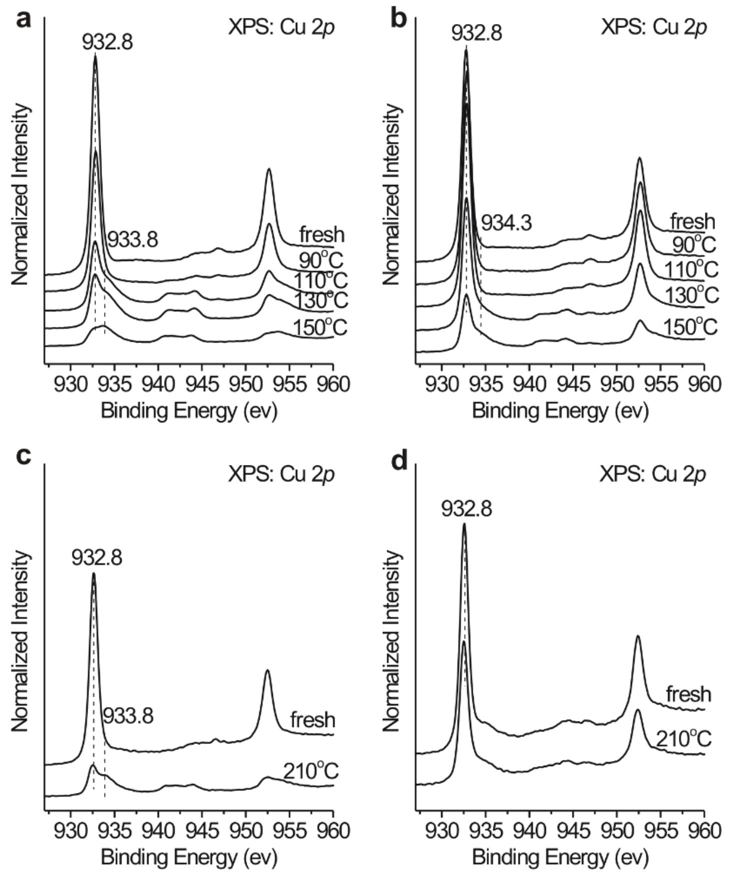

Supplementary Figure 8. Cu 2*p* XPS spectra of (**a**) c-Cu_2O-27, (**b**) c-Cu_2O-106, (**c**) c-Cu_2O-774 and (**d**) d-Cu_2O-439 NCs evaluated at different reaction temperatures.

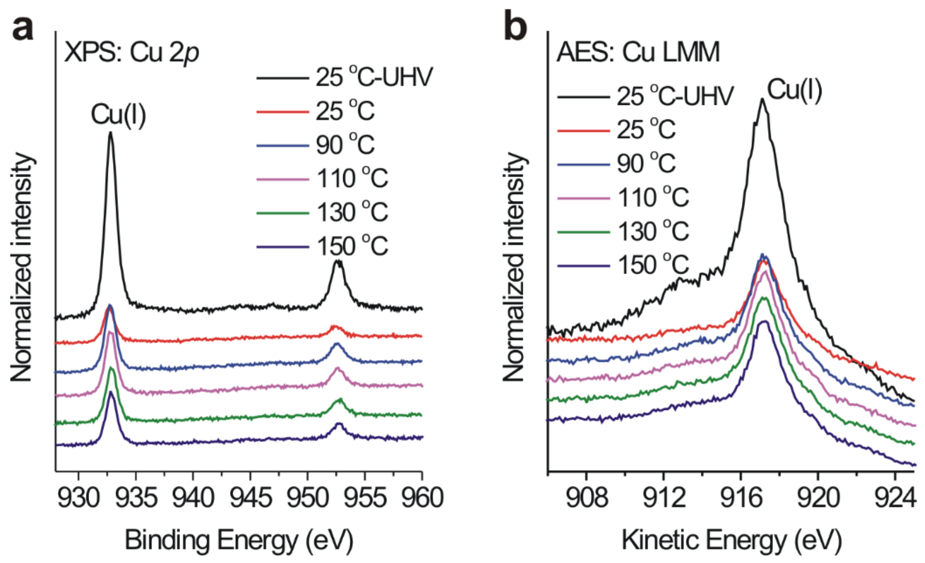

Supplementary Figure 9. (**a**) Cu 2*p* NAP-XPS and (**b**) Cu LMM NAP-AES spectra of c-Cu_2O-27 NCs under 0.6 mbar C_3H_6+ 0.3 mbar O_2 at different reaction temperatures. The used photon energy is 1150 eV. Corresponding spectra under ultrahigh vacuum condition are also shown.

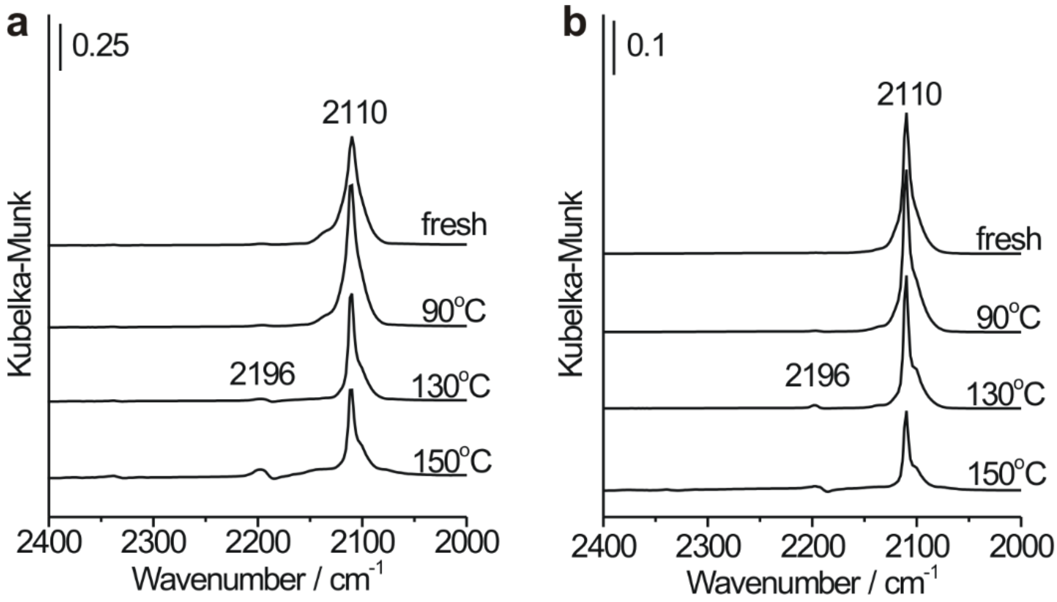

Supplementary Figure 10. In situ DRIFTS spectra of CO adsorption (P_{CO}=250 Pa) at 123 K on (**a**) c-Cu_2O-27 and (**b**) c-Cu_2O-106 subjected to C_3H_6 oxidation reaction at different temperatures.

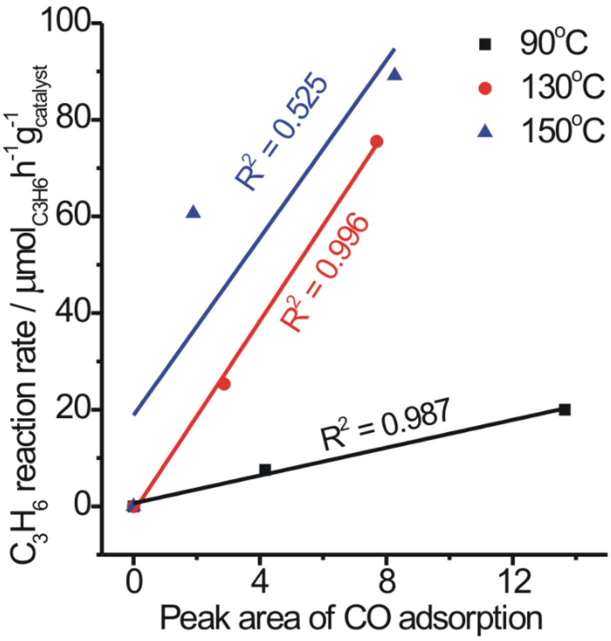

Supplementary Figure 11. C_3H_6 reaction rate of c-Cu_2O-27 and c-Cu_2O-106 at different temperature as a function of the peak area of CO adsorbed on $Cu_2O\{110\}$ edge sites of c-Cu_2O-27 and c-Cu_2O-106 derived from corresponding DRIFTS results.

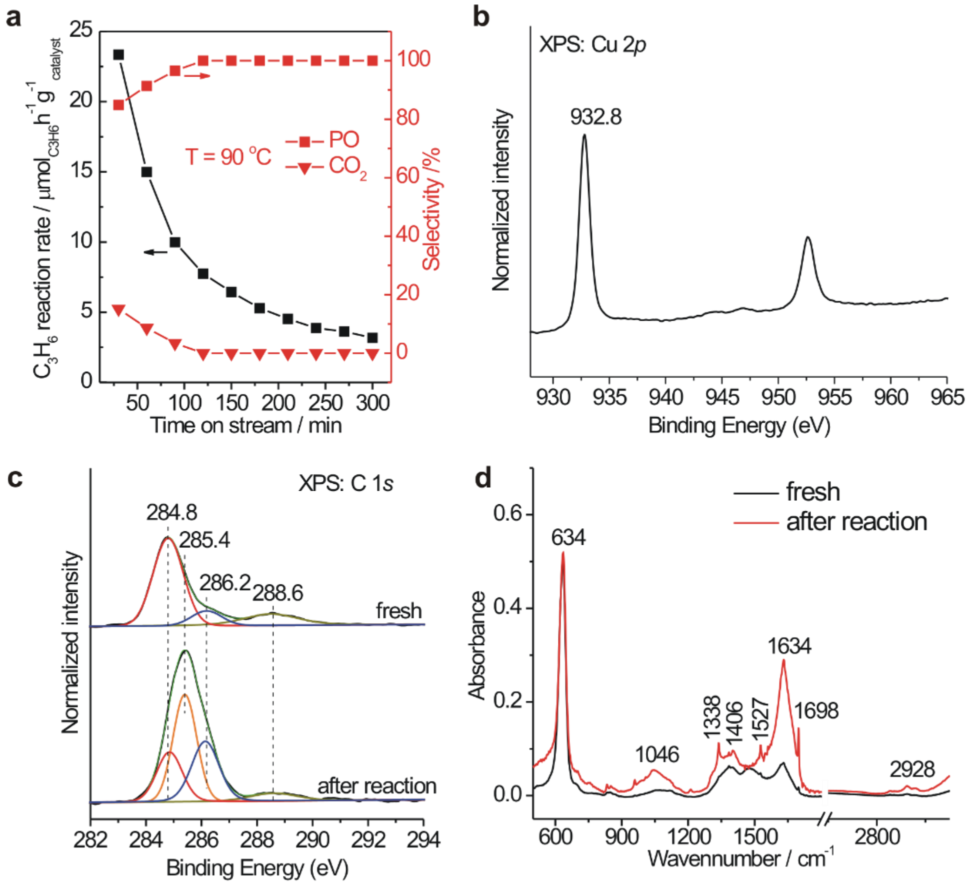

Supplementary Figure 12. (**a**) Stability of c-Cu_2O-27 in catalyzing C_3H_6 oxidation with molecular oxygen. (**b**) Cu 2*p* XPS spectra of c-Cu_2O-27 after the catalytic stability test at 90 °C. (**c**) C 1*s* XPS spectra and (**d**) Transmission infrared of c-Cu_2O-27 before and after the catalytic stability test at 90 °C.

Supplementary Table 2. Assignment of vibrational bands observed in the DRIFTS spectra.

Assignment	Wavenumber / cm^{-1}	Ref.
$\nu(CH_3)$ in C_3H_6 gas	2954	2,3
$\nu(CH_3)$ in $C_3H_6(a)_{Cu}$, $C_3H_6(a)_{Cu,O}$ & $C_3H_6(a)_{O,O}$	2925, 2860	4
ν(C-H) in HCOO(a)	2960, 2885	5
$\nu(CH_2)$ in $C_3H_5(a)_O$	2834	6
$\nu_{as}(CO_2(a))$	2338, 2190	7
ν(C=C) in C_3H_6 gas & CH_2=CH-CHO(a)	1652	2-4,8,9
ν(C=C) in $C_3H_5(a)_O$	1608	4,10
ν(C=C) in $C_3H_6(a)_{Cu}$	1590	1,11
ν(C-O) in HCOO(a)	1567, 1368	4-6,
ν(C=C) in $C_3H_6(a)_{Cu,O}$ & $C_3H_6(a)_{O,O}$	1462	1
ν_{as}(C-C-C) in $C_3H_5(a)_{Cu}$	1434	1,4
$\delta(-CH_3)$	1398	4,6
δ(C-H)	1272, 1252	1,4

Supplementary Table 3. Calculated barrier (E_a), reaction enthalpy(ΔE), and Gibbs free energy change (ΔG) of the key elementary steps for propylene oxidation to propylene oxide (PO) and acrolein on Cu_2O{110}.

Reaction step	E_a/ev	ΔE /eV	ΔG/eV
CH_3CHCH_2+*→ CH_3CHCH_2*	-	-0.53	-0.02
CH_3CHCH_2*→ PO*+V_O	1.48	1.28	1.41
O_2+V_O+*→O_2*/V_O	-	-1.65	-0.95
O_2*/V_O+*→O*+O*(lattice)	0.32	-0.98	-0.92
CH_3CHCH_2+O*→ CH_3CHCH_2O*	-	-0.56	-0.05
CH_3CHCH_2O*→ PO*	0.37	-0.98	-1.03
CH_3CHCH_2+O_2+*→ OOMP*	-	-0.34	-0.07
OOMP*+*→PO*+O*	0.68	-1.88	-1.87
CH_3CHCH_2*+*→ CH_2CHCH_2*+H*	0.75	-0.17	-0.05
CH_2CHCH_2*+O*(lattice)→ CH_2CHCHO*+H*	1.05	-0.84	-0.77
CH_2CHCHO*→ CH_2CHCHO+*+V_O	0.96	0.96	0.48

Gibbs free energy change is calculated using equation 8.

$$\Delta G = \Delta E + \Delta ZPE - T\Delta S \qquad (8)$$

The most important entropic contributions are assumed from the translational and vibrational entropies, which are calculated at 373 K and standard pressure as an example using equations 9 and 10, respectively.

$$S_{trans} = 1.5R\ln(2\pi M k_b T) - 3R\ln h + R\ln(k_b T / P) + 2.5R \qquad (9)$$

$$S_{vib} = R[\frac{hv}{k_b T}(e^{\frac{hv}{k_b T}} - 1)^{-1} - \ln(1 - e^{-\frac{hv}{k_b T}})] \qquad (10)$$

where M, R, k_b, h, T, P, and ν are molecular weight, ideal gas constant, Boltzmann constant, Planck constant, temperature, pressure, and calculated vibrational frequencies, respectively.

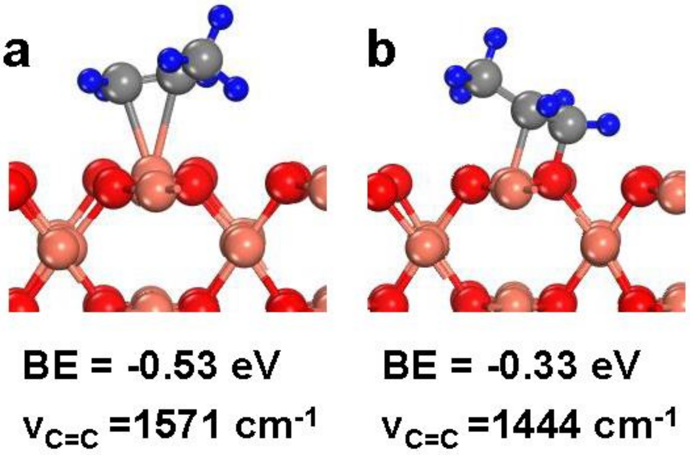

Supplementary Figure 13. Optimized geometries for propylene adsorption on $Cu_2O(110)$: (a) C_3H_6 binding to a surface Cu atom via its two carbon atoms from -CH_2 and -CH groups and (b) C_3H_6 binding to a surface Cu and a surface O atoms via its two carbon atoms from -CH_2 and -CH groups. The orange, red, gray, and blue spheres represent Cu, O, C, and H atoms, respectively.

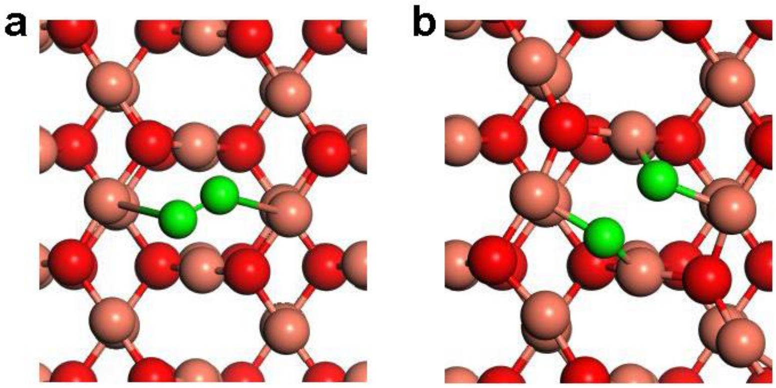

Supplementary Figure 14. Optimized structures of adsorbed O_2 (**a**) and the transition state of O_2 dissociation (**b**) on perfect $Cu_2O(110)$. The orange, green, and red spheres represent Cu, O in O_2, and O atoms, respectively.

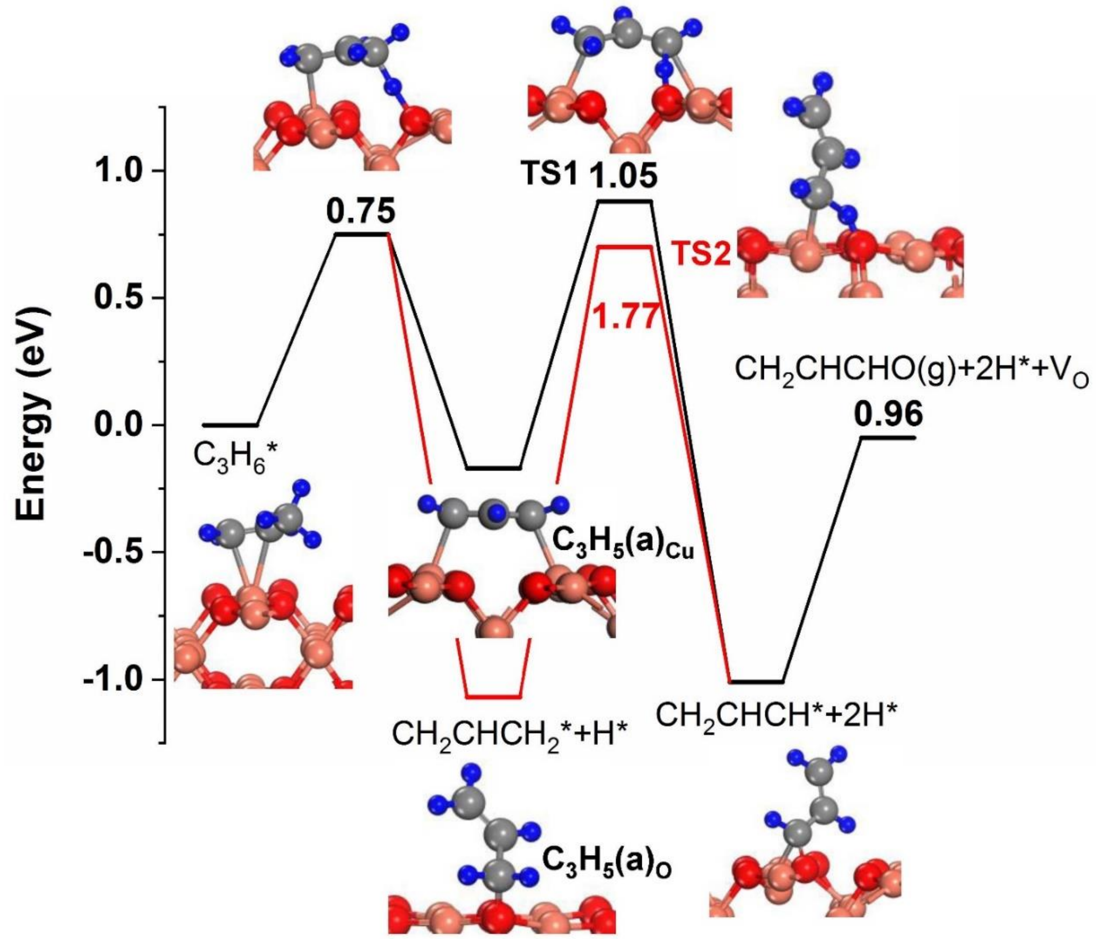

Supplementary Figure 15. Energy profile along with the optimized structures of intermediates and transition states for acrolein formation on $Cu_2O(110)$. The orange, red, gray, and blue spheres represent Cu, O, C, and H atoms, respectively.

Supplementary references

1. Hua, Q. *et al.* Crystal-plane-controlled selectivity of Cu_2O catalysts in propylene oxidation with molecular oxygen. *Angew. Chem. Int. Ed.* **53**, 4856–4861 (2014).
2. Huang, W. X. & White, J. M. Transition from π-bonded to di-σ metallacyclic propene on O-modified Ag(111). *Langmuir* **18**, 9622–9624 (2002).
3. Silvi, B., Labarbe, P., & Perchard, J. P. *Spectrochim. Acta A* **29**, 263–276 (1973).
4. Davydov, A. A., Mikhaltchenko, V. G., Sokolovskii, V. D., & Boreskov, G. K. Surface complexes of propylene and their role in catalytic oxidation. *J. Catal.* **55**, 299–313 (1978).
5. Wang, Y. et al. Exploring the ternary interactions in Cu-ZnO-ZrO_2 catalysts for efficient CO_2 hydrogenation to methanol. *Nat. Commun.* **10**, 1166 (2019).
6. Gerei, S. V., Rozhkova, E. V., & Gorokhovatsky, Y. B. Propylene and oxygen chemisorption on cupric oxide and cuprous oxide catalysts. *J. Catal.* **28**, 341–350 (1973).
7. Chen, S. *et al.* Probing surface structures of CeO_2, TiO_2, and Cu_2O nanocrystals with CO and CO_2 chemisorption. *J. Phys. Chem. C.* **120**, 21472–21485 (2016).
8. E. Finocchio, G. Busca, V. Lorenzelli, R. J. Willey, FTIR studies on the selective oxidation and combustion of light hydrocarbonsat metal oxide surfaces. *J. Chem. Soc. – Faraday Trans.* **90**, 3347 (1994).
9. Grabowski, R., Haber, J., & Sloczynski, J. IR-spectroscopic studies of the interaction of propylene and acrolein with a $CoMoO_4$ catalyst. *React. Kinet. Catal. Lett.* **12**, 119–124 (1979).
10. Wang, Y., Zheng, W., Chen, F., & Zhan, X. Mechanistic study of propylene oxidation over a Bi–molybdate catalyst by in situ DRIFTS and probe molecules. *Appl. Catal., A* **351**, 75–81 (2008).
11. Su, W., Wang, S., Ying, P., Feng, Z. & Li, C. A molecular insight into propylene epoxidation on Cu/SiO_2 catalysts using O_2 as oxidant. *J. Catal.* **268**, 165–174 (2009).
